# ST-elevation myocardial infarction from septic embolism secondary to prosthetic aortic valve endocarditis: a case report

**DOI:** 10.1093/ehjcr/ytae420

**Published:** 2024-08-09

**Authors:** Miklos Rohla, Legate Philip, Janina Wolf, Farouc A Jaffer, Lorenz Räber

**Affiliations:** Department of Cardiology, Bern University Hospital, Inselspital, University of Bern, Freiburgstrasse 20, 3010 Bern, Switzerland; Department of Cardiology, Bern University Hospital, Inselspital, University of Bern, Freiburgstrasse 20, 3010 Bern, Switzerland; Institute of Pathology, University of Bern, Bern, Switzerland; Cardiology Division, Massachusetts General Hospital, Boston, MA, USA; Harvard Medical School, Boston, MA, USA; Department of Cardiology, Bern University Hospital, Inselspital, University of Bern, Freiburgstrasse 20, 3010 Bern, Switzerland

**Keywords:** Case report, STEMI, Coronary emboli, Prosthetic valve infective endocarditis

## Abstract

**Background:**

ST-elevation myocardial infarction (STEMI) is a cardiac emergency that requires prompt diagnosis and treatment. We describe a challenging and complex case of managing acute STEMI in a patient with severe anaemia, deranged clotting profile, and infective prodrome.

**Case summary:**

A 54-year-old Caucasian gentleman was referred by his general practitioner as an emergency after presenting with acute onset of chest pain. His electrocardiogram revealed anterior ST elevation. His past medical history includes a mechanical aortic valve, requiring anticoagulation, and a recent gastrointestinal bleed secondary to type C gastritis. His initial presentation was further complicated by severe anaemia, deranged clotting profile, and elevated infective markers. He required a prompt transfer to the catheterization laboratory to assess and stabilize the situation. We discuss the emerging challenges during treatment, particularly as the diagnosis of septic embolism from infective prosthetic valve endocarditis was unfolding, requiring urgent cardiac surgery.

**Discussion:**

Acute coronary vessel closure leading to STEMI from septic embolism secondary to prosthetic aortic valve endocarditis is very rare. It is essential to consider the whole picture of the presentation for timely diagnosis and tailored treatment.

**Learning points**

- We present an extremely rare case of acute anterior myocardial infarction caused by septic embolism from a mechanical aortic valve vegetation (confirmed by histopathological evaluation of the aspirated thrombus material).
- Acute invasive and non-invasive management of this patient was complicated by severe anaemia and a hypercoagulable state
- The individualized acute care approach included aspirational thrombectomy, balloon angioplasty, and urgent surgery for prosthetic aortic valve endocarditis.

## Introduction

ST-elevation myocardial infarction (STEMI) is a cardiac emergency that requires prompt diagnosis and treatment as it often implies an acute coronary vessel closure is the cause. Timely intervention has both short- and long-term benefits. The acute management of STEMI requires restoring blood flow in the occluded vessel.^1^ With percutaneous coronary intervention (PCI), this is achieved with successful wiring of the vessel and subsequently addressing the cause of the occlusion. Although the armamentarium now includes balloons, stents, and aspiration catheters, it still requires a combination approach with pharmacological agents including anti-platelets and anticoagulants. We describe a challenging and complex case of managing acute STEMI in a patient with severe anaemia, deranged clotting profile, and infective prodrome.^1,2^

## Summary figure

**Figure:**
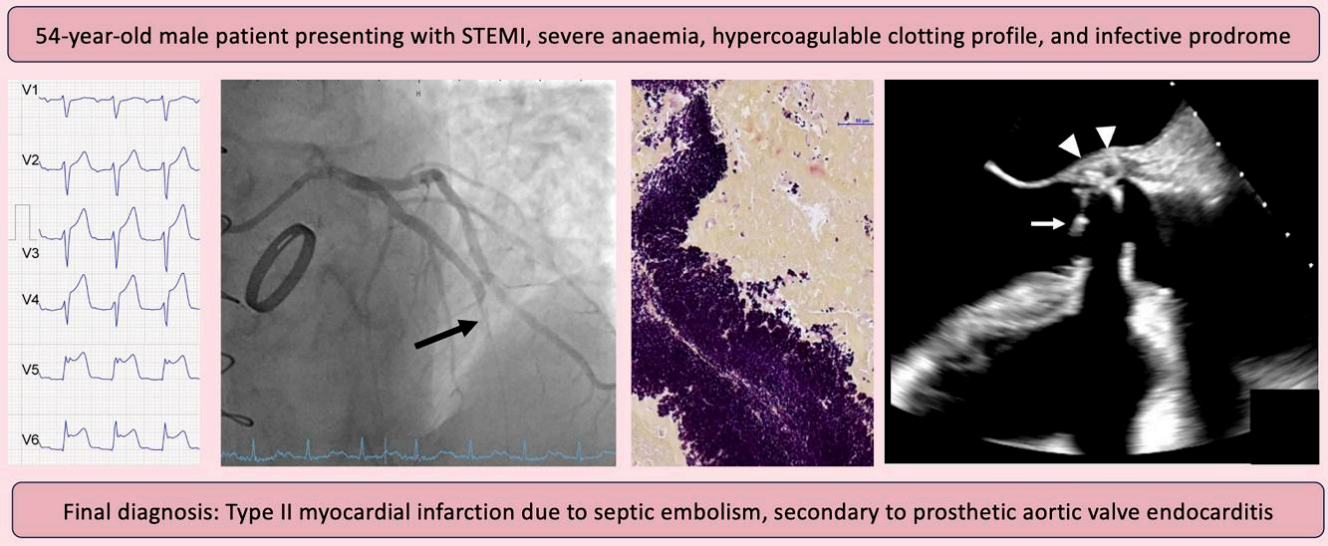


## Case presentation

**Figure 1:**
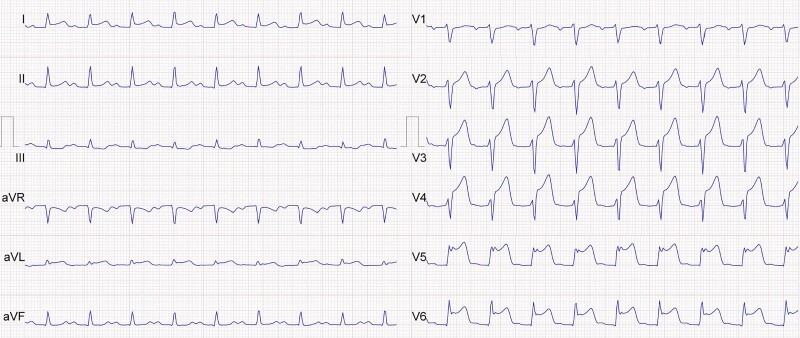
Initial electrocardiogram: the electrocardiogram shows sinus tachycardia, 1st-degree atrioventricular block, narrow QRS complexes, and significant ST-segment elevation in leads V3-V6 and I.

A 54-year-old male patient presented to his general practitioner (GP) due to acute onset of chest pain for 2.5 h. In view of the electrocardiogram (ECG) suggestive of acute anterior ST-elevation myocardial infarction (STEMI, *Figure 1*), the patient was transferred to our tertiary centre for primary percutaneous coronary intervention (PCI).

Prior to arrival, his GP provided notification that the results from the blood draw indicated the patient had severe anaemia with a haemoglobin level of 6.4 g/dL (reference range, 13–17.5 g/dL), a mean corpuscular volume of 68 fl (reference range, 82–100 fl), and a mean corpuscular haemoglobin of 20 pg (reference range, 27–34 pg). Additionally, the platelet count was above the normal range at 530 g/L (reference range, 125–350 g/L), and there was an elevation of C-reactive protein at 93 mg/L (reference value, <10 mg/L).

The patient was diverted to the emergency department for further investigations.

On physical examination, the patient appeared pale. Otherwise, the brief examination at the bedside showed no remarkable findings. The temporal temperature was 39.4°C, the heart rate 103 b.p.m., blood pressure 120/60 mmHg, respiratory rate normal, and oxygen saturation 96% in ambient air. Echocardiography showed a mildly reduced left ventricular function with apical akinesia and anteroseptal hypokinesia, and a mean transaortic pressure gradient of 20 mmHg. Following point-of-care testing, the international normalized ratio (INR) was greater than the assay and could quantify (i.e. INR > 8).

**Figure 2:**
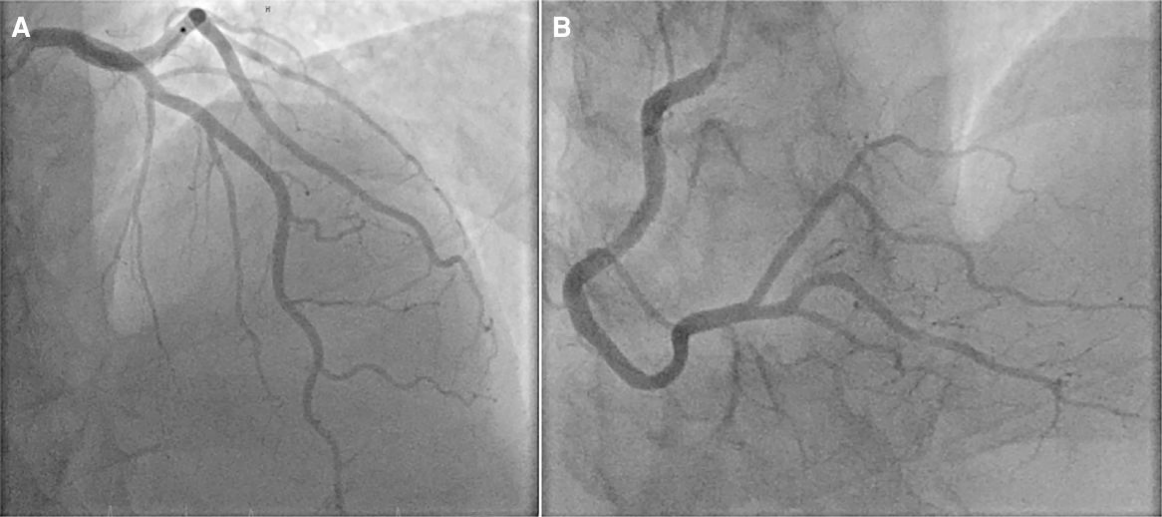
Pre-operative coronary angiogram: a coronary angiogram 3 years before, prior to aortic valve replacement, shows healthy-appearing coronaries. (A) Left coronary artery. (B) Right coronary artery.

The past medical history was notable for an aortic valve replacement with a mechanical bioprosthesis due to severe aortic stenosis 3 years prior. The coronary angiography prior to valve surgery revealed normal coronary arteries (*Figure 2*). An episode of alcohol-induced hepatitis 14 months prior was accompanied by an INR derailment (under phenprocoumon), melena, and mild anaemia (haemoglobin 12.9 g/dL). Gastroscopy showed a type C gastritis, no ulcer or acute source of bleeding, and no oesophageal varices. Cardiovascular risk factors included diabetes and dyslipidaemia.

On this admission, the patient presented with signs and symptoms consistent with acute anterior myocardial infarction (MI). Additional relevant findings were a pronounced microcytic hypochromic anaemia, severe INR derailment on treatment with phenprocoumon, and signs of infection. Our approach to this case was as follows.

### Differential diagnosis for myocardial infarction

The most frequent aetiology for STEMI is type 1 MI (89% in a ‘real-world’ scenario).^3^ According to the 4th universal definition, type I MI is caused by atherothrombotic coronary artery disease (CAD), precipitated by atherosclerotic plaque disruption.^4^

Owing to the clinical circumstances, type II MI, which is broadly characterized by an oxygen supply/demand mismatch, had to be considered. Coronary aetiologies of type II MI include spontaneous coronary artery dissection, coronary intramural haematoma, coronary spasm, coronary embolism, and microvascular dysfunction. Non-coronary causes include an even more heterogeneous set of conditions such as severe hypotension/hypertension, bradyarrhythmias or tachyarrhythmias, hypoxemia, or severe anaemia. Myocarditis and Takotsubo syndrome also fall into this category.^1,5^

In the present case, the pattern of ECG changes support a coronary aetiology rather than global ischaemia secondary to anaemia and/or bleeding. Echocardiography is key to promptly establish a differential diagnosis: Myocarditis rarely presents with anterior wall motion abnormalities, and in Takotsubo cardiomyopathy, basal segments appear hypercontractile, while the apex is often dilated.

The clinical scenario supported the diagnosis of acute type II MI with an underlying coronary cause.

### Differential diagnosis for anaemia

Upon considering the differential diagnosis for anaemia, several potential causes were taken into account. Although the INR derailment might suggest active bleeding, anaemia due to acute blood loss is usually normochromic and normocytic, except for chronic bleeding and subsequent iron depletion. The most likely cause for microcytic hypochromic anaemia is iron deficiency, either through insufficient dietary intake, chronic blood loss, or increased demand. In this patient, chronic blood loss in association with the previously diagnosed erosive gastritis, potentially aggravated by oral anticoagulation, appear likely. Additionally, hypoproliferative anaemia in response to systemic illness or inflammation could be considered. Other rare causes such as disseminated intravascular coagulation or thrombotic thrombocytopenic purpura appeared less likely in the presence of elevated platelet count.

### Differential diagnosis for infection

The patient did not report respiratory symptoms, urogenital symptoms, recent dental procedures, or skin manifestations. Bedside echocardiography did not reveal large vegetations on the mitral and tricuspid valves; the mechanical prosthesis could not be assessed sufficiently due to imaging conditions.

Owing to the clinical circumstances, the working diagnosis of an acute left anterior descending artery (LAD) occlusion secondary to a septic embolism was developed.

**Figure 3:**
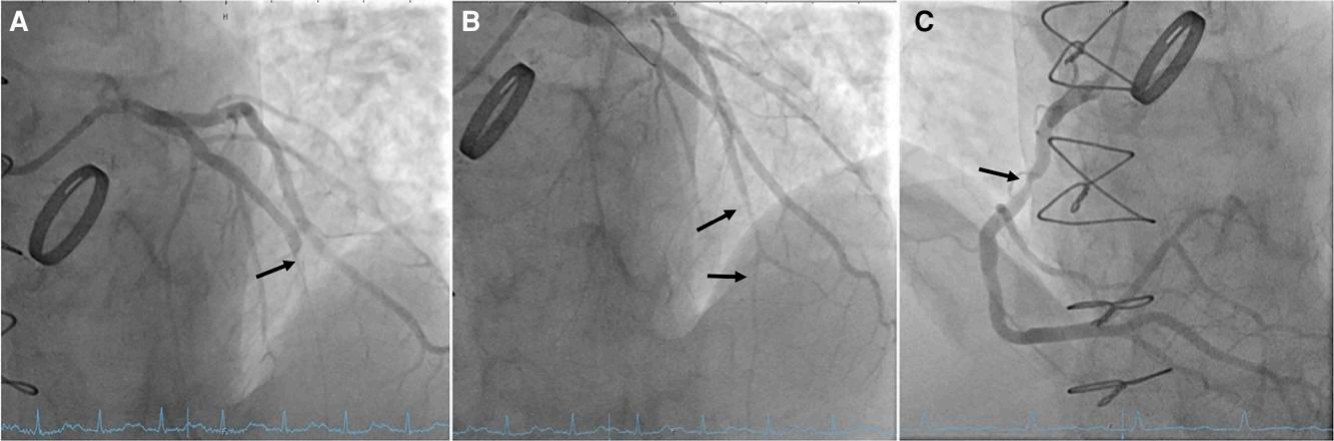
Acute coronary angiogram and percutaneous coronary intervention: the acutely performed coronary angiogram shows an occluded mid left anterior descending artery (A). Thrombolysis in myocardial infarction flow grade III could be achieved after repetitive thrombectomies and balloon angioplasties (B). There was a disease progression in the right coronary artery, with significant stenosis of the mid segment (C).

Acute coronary angiography through a radial access revealed an occlusion of the mid LAD and a significant stenosis of the mid right coronary artery (*Figure 3*; Supplementary material online, *Videos S1* and *S2*). As the angiography was suggestive of an embolic origin (blunt occlusion of a minimally diseased vessel), repeated aspirational thrombectomies and percutaneous transluminal coronary angioplasty (PTCA) with balloon dilatations were performed, re-establishing thrombolysis in myocardial infarction grade III flow. The door-to-balloon time was 58 min. Given the severe anaemia, INR derailment, and the acceptable angiographic result, stent implantation was withheld. The aspirated material was sent for histological examination. Cinefluoroscopy of the mechanical valve showed normal leaflet opening.

Post-procedurally, the patient received a total of three units of packed red blood cells, vitamin K infusion following unfractionated heparin once INR reached a value <3.5, gastric protection, and empiric antibiotics. Antiplatelet drugs were initially withheld.

The maximum creatine kinase (CK) and high-sensitivity troponin T levels were 1297 U/L and 6680 ng/L the day after admission, respectively.

**Figure 4:**
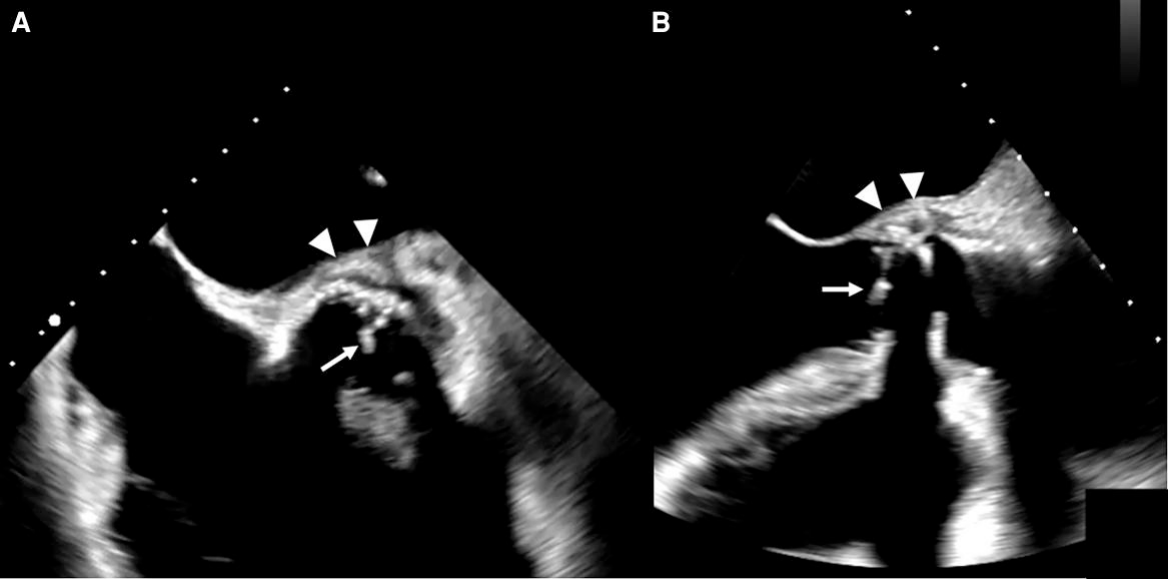
Transoesophageal echocardiography: transoesophageal echocardiography shows ring abscess [(A) short-axis view and (B) mid-oesophageal long-axis view, arrow heads] and a mobile vegetation on the mechanical aortic valve prosthesis prolapsing into the left ventricular outflow tract [(A and B), arrows].

Transoesophageal echocardiography (TOE) revealed one 0.7 cm non-mobile mass in-between the mechanical valve leaflets and another 1 cm mobile vegetation attached to the valve ring at the level of the coronary sinus (*Figure 4*; Supplementary material online, *Video S3*). There was moderate (eccentric) paravalvular regurgitation and suspected abscess of the entire ring. The mitral and tricuspid valves showed moderate regurgitation. According to institutional guidelines, empiric antibiotic treatment with 2.2 g amoxicillin and clavulanic acid i.v. six times daily was started.

**Figure 5:**
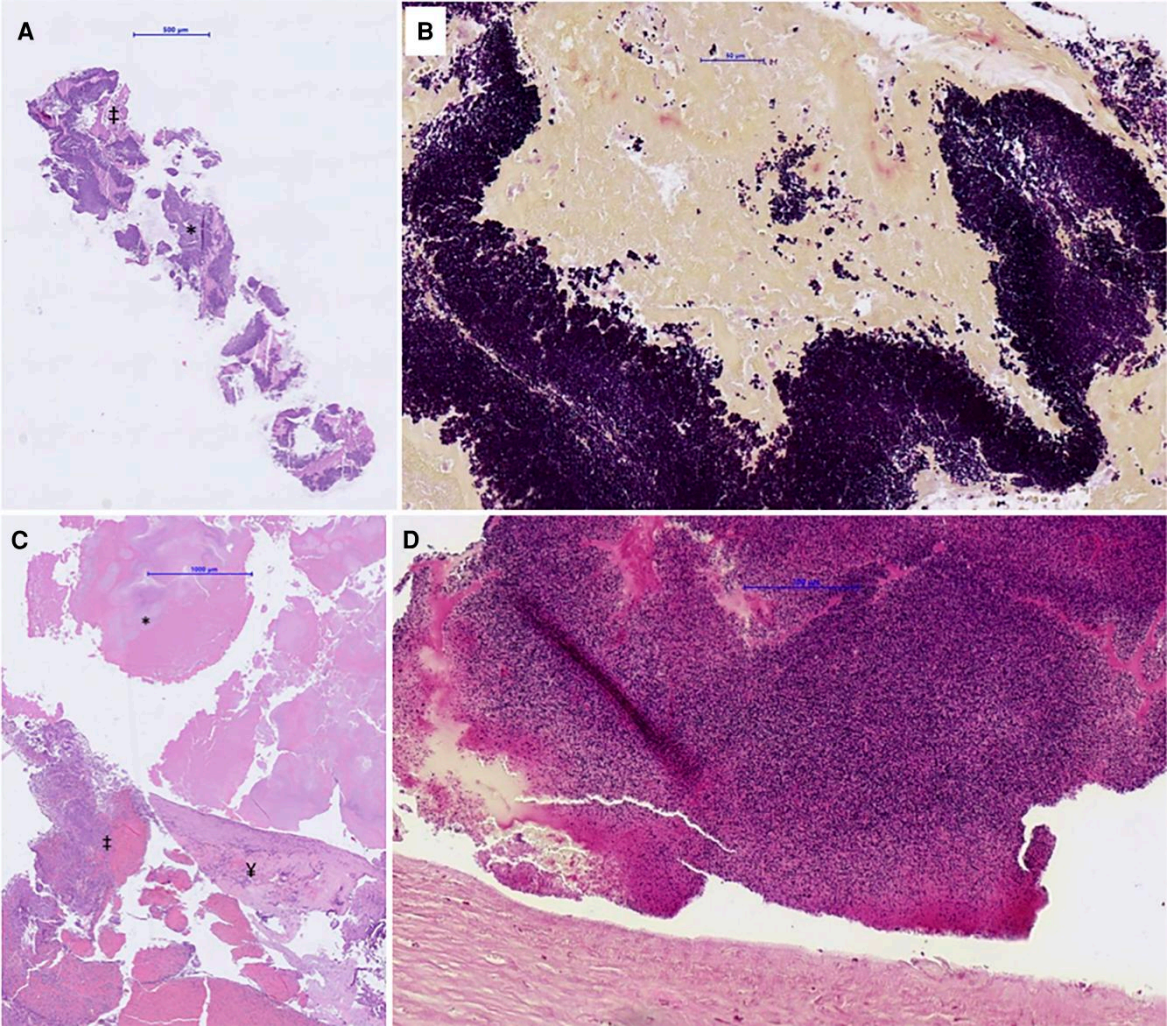
Pathological study of the aspirated material and aortic annulus: aspirated material from the left anterior descending artery (A) shows fibrin (‡) with colonization of bacteria (*, 4 × magnification). Gram-staining demonstrates extensive evidence of Gram-positive cocci, staining deep purple [(B), 40 × magnification]. Material of the native aortic annulus (C) shows collagenous parts of the valve (¥), blood and inflammatory cells (‡), and fibrin with extensive bacterial colonization (*, 2 × magnification). Gram-positive cocci, staining deep purple, can be visualized upon 20 × magnification (D).

Blood cultures revealed a positive result for *Aerococcus urinae*. Histopathological examination of the aspirated thrombus revealed abundant Gram-positive cocci (*Figure 5*).

Replacement of the mechanical aortic prosthesis, single saphenous vein bypass to the right coronary artery, and reconstruction of the mitral and tricuspid valves were planned. Visual and eventually histological inspection of the prosthetic valve confirmed abscess of the valve ring (*Figure 5*).

The patient was discharged on the 7th post-operative day for cardiac rehabilitation with a good post-operative result on transthoracic echocardiography; stable normochromic, normocytic anaemia (Hb 8.3 g/dL); and low levels of CRP (41 mg/L). Antibiotic treatment was discontinued at discharge.

After cardiac rehabilitation, the patient could regain a normal cardiorespiratory fitness (6 min walking distance of 600 m) and remain free from cardiac symptoms.

## Discussion

The patient presented with a rare case of 4th universal definition type II MI due to septic embolism secondary to prosthetic aortic valve endocarditis.^4^

The initial management of this patient is challenging, not only due to complicating conditions, but because decision-making needs to be performed in a timely manner. Avoiding stent implantation and subsequent escalation of antithrombotic therapy appeared to be a reasonable approach and was associated with little enzymatic and echocardiographic myocardial damage.

Equally important is the treatment of the underlying disease, i.e. infective prosthetic valve endocarditis. The recent 2023 guidelines of the European Society of Cardiology on the management of endocarditis recommend surgery either in the presence of heart failure (caused by severe regurgitation or obstruction), uncontrolled infection (including abscess, false aneurysm, fistula, or enlarging vegetation), or embolism (either high risk for or established embolism, mostly in the presence of vegetations ≥ 10 mm).^2^ Ring abscess, a high risk for recurrent embolism, low likelihood for eradication of the biofilm-forming pathogen, and low surgical risk in a young patient support an urgent re-do surgery.

*Aerococcus urinae*, a Gram-positive bacterium, is a rarely reported pathogen, partly because it can be misclassified as an alpha-haemolytic streptococcus due to its growth characteristics and haemolysis pattern. Patients infected with *A. urinae* are often elderly males who initially present with urinary tract infections. Review of the pre-operative CT showed perinephric stranding, parenchymal enlargement, and distension of the left ureter, consistent with an asymptomatic and unobstructed ascending urinary tract infection (see Supplementary material online, *Figure S1*).^6^ Like other pathogens that cause infective endocarditis (e.g. *Staphylococcus aureus* and *Enterococcus faecalis*), *A. urinae* can activate platelets through bacterial bound fibrinogen, specific immunoglobin G (IgG) bound to the bacterial surface and complement activation.^6^

The most likely cause for severe microcytic anaemia in this patient was iron deficiency, as confirmed by a transferrin saturation of 19% (reference range 20–50%). Subacute and/or chronic subclinical gastrointestinal blood loss under VKA therapy and endocarditis might have contributed to both, iron deficiency, and anaemia.

After cardiac rehabilitation, the patient could regain a normal cardiorespiratory fitness (6 min walking distance of 600 m) and was free from cardiac symptoms.

## Lead author biography


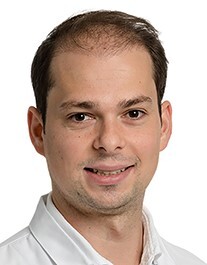


Dr Rohla holds a position as invasive attending physician at Inselspital, University Hospital Bern. His research focuses around medical and invasive treatments of acute coronary syndromes, antithrombotic therapies in patients with coronary artery disease or atrial fibrillation, and control of risk factors, particularly arterial hypertension. Main clinical areas of interest are interventional cardiology and intensive care medicine.

## Supplementary Material

ytae420_Supplementary_Data

## Data Availability

The data underlying this article are available in the article and in its online Supplementary material.

## Supplementary material

**Consent:** The authors confirm that written informed consent for submission and publication of this case report, including images and associated text, has been obtained from the patient in line with the COPE guidelines.

**Funding:** None declared.
