# Supplementary material for: ST-elevation myocardial infarction from septic embolism secondary to prosthetic aortic valve endocarditis: a case report
Source: Eur Heart J Case Rep. 2024 Aug 9;8(8):ytae420. doi: 10.1093/ehjcr/ytae420 (PMC11337003; doi:10.1093/ehjcr/ytae420)
Supplement: ytae420_Supplementary_Data [file ytae420_supplementary_data.zip › SUPPLEMENTAL MATERIAL.docx]

**SUPPLEMENTAL MATERIAL**

**SUPPLEMENTAL FIGURES**

**
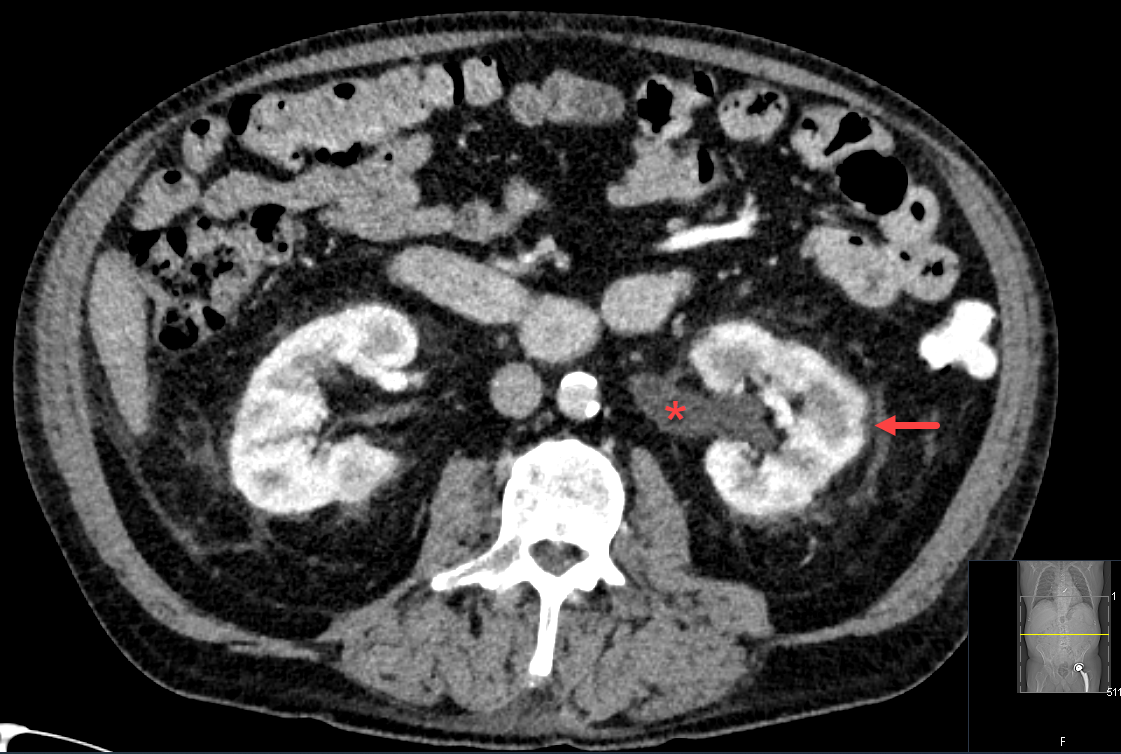
**

**Figure S1: Computed tomography for detection of further septic emboli:**

The computed tomography performed pre-operatively shows signs of left-sided pyelonephritis with marked distension of the left renal pelvis and ureter (*) and perinephritic stranding (arrow).
